# Quantitative Intersectionality Scoring System (QISS): Opportunities for Enhancing Predictive Modeling, Comparative Analysis, Health Needs Assessment, and Policy Evaluation

**DOI:** 10.31586/jsmhes.2024.1066

**Published:** 2024-10-19

**Authors:** Shervin Assari, Hossein Zare

**Affiliations:** 1 Department of Internal Medicine, Charles R. Drew University of Medicine and Science, Los Angeles, CA, United States; 2 Department of Family Medicine, Charles R. Drew University of Medicine and Science, Los Angeles, CA, United States; 3 Department of Urban Public Health, Charles R. Drew University of Medicine and Science, Los Angeles, CA, United States; 4 Marginalization-Related Diminished Returns (MDRs) Center, Los Angeles, CA, United States; 5 Department of Health Policy and Management, Johns Hopkins Bloomberg School of Public Health, Baltimore, MD, United States; 6 School of Business, University of Maryland Global Campus (UMGC), Adelphi, MD, United States

**Keywords:** Intersectionality, Race, Ethnicity, Gender, Sex, Class, Sexual Orientation

## Abstract

Intersectionality has significantly enhanced our understanding of how overlapping social identities—such as race, ethnicity, gender, sex, class, and sexual orientation—interact to shape individual experiences. However, despite its theoretical importance, much of the existing literature has relied on qualitative approaches to define and study intersectionality, limiting its application in predictive modeling, comparative analysis, and policy development. This paper introduces the concept of Quantitative Intersectionality Scoring System (QISS), a novel approach that assigns numerical scores to intersecting identities, thereby enabling a more systematic and data-driven analysis of intersectional effects. We argue that QISS can substantially enhance the utility and predictive validity of quantitative models by capturing the complexities of multiple, overlapping social determinants. By presenting concrete examples, such as the varying impacts of socioeconomic mobility on life expectancy among different intersectional groups, we demonstrate how QISS can yield more precise and reliable forecasts. Such a shift would allow policymakers and service providers to dynamically assess economic and health needs, as well as the uncertainties around them, as individuals move through different social and economic contexts. QISS-based models could be more responsive to the complexities of intersecting identities, allowing for a more quantified and nuanced evaluation of policy interventions. We conclude by discussing the challenges of implementing QISS and emphasizing the need for further research to validate these quantifications using robust quantitative methods. Ultimately, adopting QISS has the potential to improve the accuracy of predictive models and the effectiveness of policies aimed at promoting social justice and health equity.

## 1. Introduction

Intersectionality has fundamentally reshaped our understanding of how overlapping and interdependent social identities—such as race, ethnicity, gender, sex, class, and sexual orientation—interact to create unique experiences of privilege and discrimination. Developed initially as a theoretical framework by Kimberlé Crenshaw in 1989, intersectionality was designed to critique the limitations of single-axis analyses of discrimination that often failed to account for the compounded experiences faced by individuals who occupy multiple marginalized identities. Since its inception, intersectionality has been widely adopted across various fields, including law, sociology, public health, psychology, and education, providing valuable insights into the complex ways that systems of power and oppression overlap. In public health, for example, intersectionality has been instrumental in understanding why certain groups, such as Black women or lesbian, gay, bisexual, transgender and queer (LGBTQ+) communities, face disproportionate health disparities. These insights have proven vital for addressing the multifaceted determinants of health outcomes and developing more comprehensive approaches to health equity.

Despite its significant contributions, much of the literature on intersectionality has been predominantly qualitative, focusing on descriptive analyses that explore how different social identities intersect and how these intersections shape experiences of inequality. While qualitative approaches have provided rich contextual understandings, they often fall short when applied to predictive modeling and policy development, where a more quantitative approach is needed to make accurate forecasts and inform interventions. This gap limits our ability to quantify how specific intersecting identities—such as those defined by race, gender, and socioeconomic status—affect life outcomes across different contexts. Without a robust quantitative framework, our predictive models lack the precision needed to guide data-driven policies and equitable resource allocation effectively.

This paper introduces the concept of Quantitative Intersectionality Scoring System (QISS) as a novel method to bridge this gap by providing a systematic, numeric scoring system for intersecting identities. QISS offers a more data-driven approach to analyzing intersectional effects, allowing for the integration of intersectionality into predictive models, comparative analyses, health needs assessments, and policy evaluations. By assigning numerical scores to different combinations of intersecting identities, QISS aims to capture the compounded and multiplicative effects of social determinants, offering a more nuanced and accurate understanding of social inequalities.

The paper is organized into several sections. First, we review the Origins and Evolution of Intersectionality, providing a historical overview from its beginnings in legal studies to its expansion into public health and other disciplines. This section underscores the limitations of the qualitative approach traditionally employed and sets the stage for the need for quantitative innovation. Following this, we introduce the concept of Quantitative Intersectionality Scoring System (QISS), outlining its development, methodological approaches, and potential applications. We delve into the Development of QISS, detailing how this framework can be constructed using empirical data and statistical modeling to quantify intersectional identities and their compounded effects on various outcomes, such as health and economic stability. Next, we discuss the Methodological Approaches for Calculating QISS, including statistical techniques like regression analysis, machine learning, multi-level modeling, latent class analysis, and structural equation modeling, to provide accurate estimates of intersectionality’s impact.

The Applications of QISS in Predictive Modeling and Policy Development section explores how QISS can enhance the predictive utility of models across different domains. This section includes examples from health research, where QISS can identify which intersectional groups are most at risk for diseases or have the greatest need for specific interventions. It also covers potential policy applications, illustrating how QISS can optimize resource allocation and program design. Following this, we present two innovative approaches to operationalizing intersectionality in research—Array Analytical Approach and Factorial Designs. The Array Analytical Approach provides a multi-dimensional framework to systematically analyze the interactions between various intersectional identities, while Factorial Designs offer a method for examining the combined effects of multiple intersecting identities, providing more nuanced insights into how these interactions affect outcomes.

The paper then addresses the Challenges and Considerations of implementing QISS, recognizing potential limitations, ethical concerns, and the need to balance quantitative and qualitative methods to preserve the depth and complexity of intersectionality. We discuss the potential for misinterpretation or misuse of QISS and propose strategies for mitigating these risks, including interdisciplinary collaboration and community engagement. Finally, the Future Research Directions section calls for the development of more refined tools and models that quantify intersectional identities’ interactive and multiplicative effects, along with dynamic models that account for changes over time and across contexts.

In the conclusion section, we argue that the integration of QISS into intersectionality research marks a significant step forward in creating more precise, data-driven models that can better inform policy interventions aimed at reducing social inequalities. By providing a quantitative framework for intersectionality, QISS has the potential to enhance our understanding of social determinants of health, improve the accuracy of predictive models, and lead to more targeted and equitable policies.

## 2. Intersectionality

### 2.1. Definition

Intersectionality is a theoretical framework that explores how different social identities—such as race, gender, class, sexual orientation, and others—intersect and overlap, creating unique experiences of discrimination and privilege. Rather than treating these identities as isolated or additive, intersectionality emphasizes the interconnectedness of these factors and how they collectively shape an individual’s experience within society [1].

### 2.2. Brief History

The concept of intersectionality was first coined by Kimberlé Crenshaw [1], a legal scholar, in 1989. Crenshaw introduced the term in her seminal paper *Demarginalizing the Intersection of Race and Sex*^1^, where she critiqued the limitations of antidiscrimination law in addressing the experiences of Black women. She argued that the legal system often failed to recognize the unique challenges faced by individuals who occupy multiple marginalized identities, such as being both Black and a woman. Crenshaw’s work highlighted that legal cases tended to address race and gender discrimination separately, which obscured the specific forms of oppression encountered by those at the intersection of these identities. Her concept of intersectionality has since become foundational in legal studies, particularly in areas related to civil rights, feminist theory, and critical race theory [2].

### 2.3. Brief History in Public Health

The application of intersectionality in public health began in the late 1990s, as scholars recognized the need to address the complex social determinants of health that disproportionately affect marginalized populations. Public health researchers [2–8] began to use intersectionality to better understand how multiple forms of inequality—such as racism, sexism, and classism—interact to produce health disparities [2].

Intersectionality has been particularly influential in studies examining health outcomes among women of color and LGBTQ+ communities. For example, researchers have used intersectionality to explore why Black women experience higher rates of maternal mortality compared to their White counterparts [9–11], or why LGBTQ+ individuals face significant barriers to accessing healthcare [12]. By considering the overlapping social determinants that contribute to health outcomes, intersectionality has enabled a more comprehensive approach to addressing health inequities.

### 2.4. Expansion into Other Fields

Since its inception in law and its adoption in public health, intersectionality has expanded into various other disciplines, including sociology, education, psychology, and social work. In each of these fields, intersectionality is used to explore how systems of power and oppression operate across different contexts and impact individuals’ lives in multifaceted ways.

In education, for example, intersectionality has been used to examine how race, gender, and socioeconomic status affect students’ academic outcomes and access to opportunities. In sociology, it has informed analyses of how social structures perpetuate inequality across different axes of identity.

**Figure 1.**
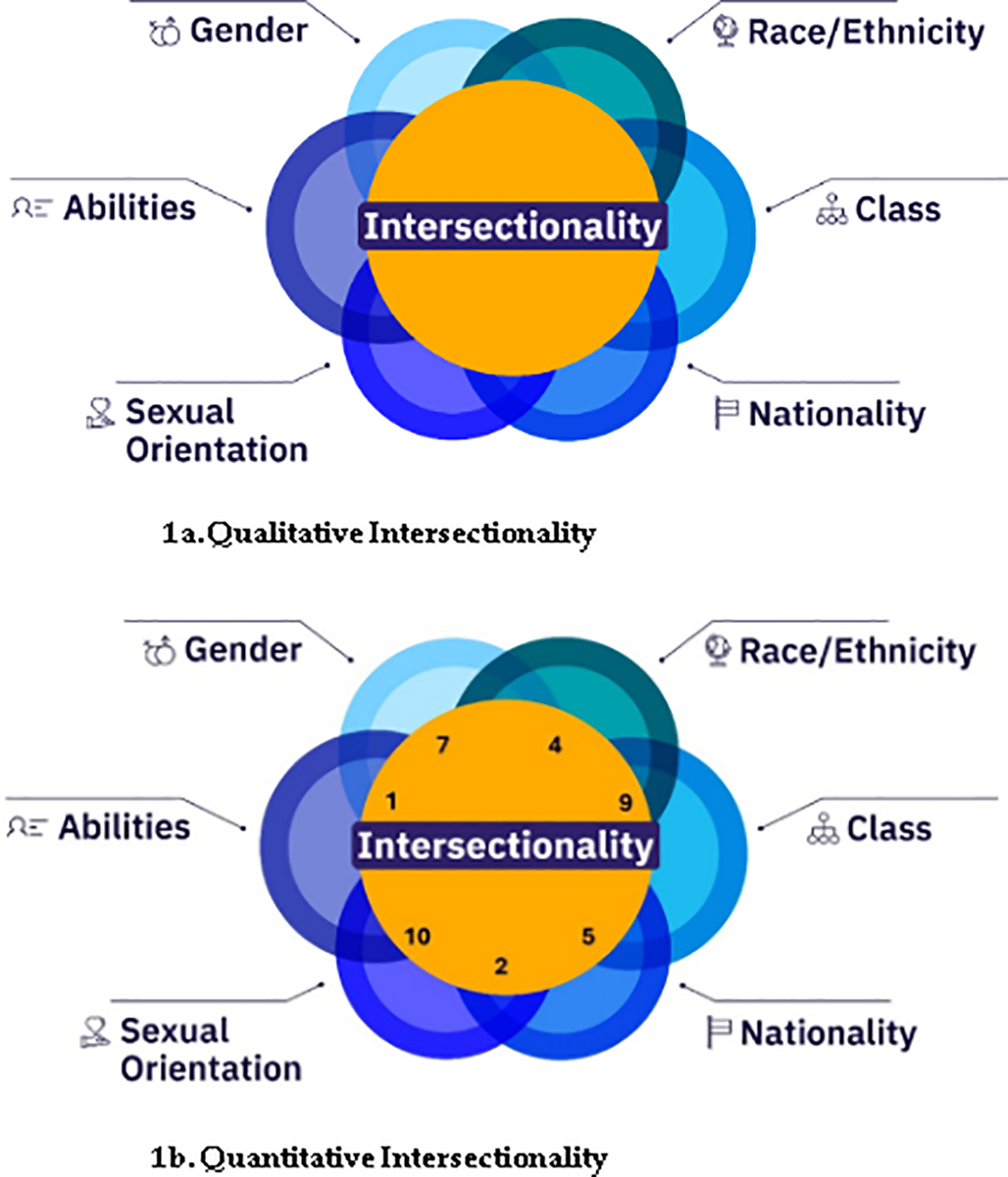
Qualitative vs. Quantitative Approach to Intersectionality

In the framework illustrated in Figure 1a, we see that social identities do not exist in isolation [13]; rather, they intersect, creating complex layers of experience and discrimination. No individual is defined solely by a single characteristic [14], such as being Black or being a woman. Instead, people experience their identities as a combination of various factors, each influencing the others.

This is exemplified in the differences between Black men’s studies and Black feminist perspectives. Black men’s studies focus on the unique challenges faced by Black men [15–19], particularly in relation to their interactions with the police and the justice system, the disproportionate impact of HIV, the portrayal of Black men in the media, their participation in crime and gang activities, and the societal aggression directed toward them. These studies emphasize the specific vulnerabilities and stigmatizations that arise from being both Black and male.

On the other hand, Black feminist perspectives highlight the compounded discrimination that Black women face, particularly in the labor market, where they often encounter both racial and gender bias. These studies also discuss the challenges associated with single motherhood, as well as the societal expectations surrounding the “strong Black mother” figure, which can impose additional burdens on Black women.

While both perspectives offer valuable insights into the distinct experiences of Black men and women, they often fall short in providing a quantitative framework that could inform predictive models [20,21], needs assessments, or policy-making. Neither approach adequately quantifies [9,22–25] how these intersecting identities affect life outcomes, nor do they offer concrete suggestions for how these insights should be integrated into predictive models or used to guide policy interventions. The need for a more comprehensive, quantifiable understanding of intersectionality is critical to developing policies that address the specific and evolving needs of individuals at these intersections of identity [26,27].

Intersectionality has fundamentally reshaped how we understand the overlapping and interdependent systems of discrimination and privilege. However, despite the rich theoretical insights it offers, much of the intersectionality literature has remained largely qualitative. While qualitative approaches provide deep contextual understanding, they often fall short when it comes to generating predictive models that can inform policies and interventions at scale. The lack of a quantitative approach in intersectionality means that we are unable to accurately predict how specific intersecting identities, such as race, gender, and socioeconomic status, impact life outcomes in different contexts.

### 2.5. The Gap in Predictive Modeling

Currently, without a robust quantitative framework for intersectionality, our predictive models remain inadequate. For instance, we lack the ability to precisely estimate what happens to the life expectancy of a male non-Latino Black person living in New York City when he transitions from a low-paying job to a high-paying one. This gap in understanding is critical because it prevents us from making informed comparisons across different contexts and populations. How does this change in socioeconomic status affect life expectancy differently for this individual compared to, say, a White woman in rural Ohio making the same economic transition? [28]

### 2.6. Example

Consider two individuals: one is a male non-Latino Black person living in New York City, and the other is a White woman living in a rural area of Ohio. Both individuals make a significant economic transition, moving from a low-paying job to a high-paying one. Traditional models might predict similar improvements in life expectancy for both, based on the assumption that higher income universally leads to better health outcomes. However, qualitative research suggests that the impact of this transition can be vastly different depending on their intersecting identities and contexts.

For the male non-Latino Black individual in New York, the intersection of race, gender, and urban living may mean that the benefits of increased income are mitigated by factors such as persistent racial discrimination, exposure to violence, or stress from navigating predominantly White workspaces. On the other hand, the White woman in Ohio may experience different challenges and advantages related to her gender and rural context, such as gender-based discrimination in the workplace or limited access to healthcare, which also influence the outcome [29–32].

For example, in the scenario above, a model using quantitative intersectionality might reveal that the life expectancy gain for the male non-Latino Black individual in New York is significantly less than that for the White woman in Ohio, despite both experiencing the same economic improvement. This insight could then inform targeted interventions to address the specific barriers faced by each group, such as anti-discrimination policies or community health initiatives.

Without a quantitative intersectionality approach, we cannot effectively model or predict these nuances. Assigning quantitative scores to intersecting identities allows us to build models that better reflect the reality of how different factors interact. For instance, a quantitative model could assign different weights to race, gender, and location, enabling us to predict more accurately how each person’s life expectancy would change with the same economic advancement.

Figure 1b presents a hypothetical depiction of the quantitative conceptualization of intersectionality. In this model, each intersectional group is assigned a single quantitative score that reflects their unique intersectional marginalization, cumulative exposure to disadvantage, and life history. Importantly, this score is not merely additive; it captures the multiplicative and interactive effects of intersecting identities, illustrating how combined factors contribute to a group’s overall marginalization. The numbers used in this figure are hypothetical and are intended to demonstrate how this approach can quantify the complex realities of intersecting identities, providing a more nuanced understanding of social inequities.

### 2.7. The Added Value of Quantitative Intersectionality

Quantitative intersectionality would allow us to move beyond broad, generalized predictions to more precise, individualized forecasts. By assigning scores to intersecting identities—such as race, gender, socioeconomic status, and geographic location—we can enhance the predictive power of our models. This approach enables us to understand not just whether a change in income leads to improved life expectancy, but how much it improves and under what conditions.

### 2.8. Dynamic Policy Implications: Assigning Needs, Uncertainty, and Predicted Productivity Scores

A critical implication of adopting a quantitative intersectionality approach is its potential to revolutionize policy design and implementation. With the ability to assign a dynamic score to individuals based on their intersecting identities and changing contexts, policymakers and service providers can better estimate the needs, uncertainties, and predicted productivity of individuals as they move through different social and economic spheres.

These scores would not be static; instead, they would adjust as the individual progresses on their social ladder or changes contexts. For example, as the male non-Latino Black individual in New York moves from a low-paying job to a high-paying one, his score would dynamically reflect not only the potential improvements in life expectancy but also the ongoing challenges and uncertainties he might face due to persistent systemic barriers. This dynamic scoring system would enable more tailored and responsive policies, ensuring that resources and support are allocated in a way that truly meets the evolving needs of individuals.

Moreover, by incorporating predicted productivity scores into this model, we can better forecast the potential contributions of individuals to the economy or society, factoring in the specific challenges they face due to their intersecting identities. This approach would allow for more equitable opportunities for advancement, as policies could be designed to maximize individuals’ potential while also addressing the systemic factors that may hinder their progress.

### 2.9. Challenges and Considerations

While the quantitative treatment of intersectionality offers significant potential for advancing research and policy, it is not without challenges and considerations. One of the primary challenges is the potential resistance from those who argue that reducing intersectionality to numbers risks oversimplifying complex social identities and experiences. Critics may contend that quantifying intersectionality could lead to viewing people and groups as mere statistics, thereby undermining the original intent of the intersectionality framework, which was to move away from such reductive, simplistic approaches.

The intersectionality movement was born out of a need to recognize and address the nuanced and multifaceted ways in which different forms of oppression and privilege intersect. By translating these complexities into quantitative terms, there is a risk of losing the rich, contextual understanding that qualitative approaches provide. Critics may worry that this shift could dilute the power of intersectionality as a tool for social justice, reducing individuals’ lived experiences to numbers and potentially reinforcing the very systems of oppression the framework seeks to dismantle.

To address these concerns, it is crucial to approach the quantitative treatment of intersectionality with care and sensitivity. Researchers must ensure that their models do not strip away the complexity and humanity of the individuals they aim to represent. This requires a balanced approach that combines the strengths of both qualitative and quantitative methods, allowing for a more comprehensive understanding of how intersecting identities influence life outcomes.

Moreover, it is essential to engage with communities and stakeholders throughout the research process to ensure that the quantitative tools developed are reflective of and responsive to their needs and experiences. By doing so, we can mitigate the risk of oversimplification and ensure that the quantitative treatment of intersectionality enhances rather than diminishes the framework’s capacity to drive meaningful social change.

In summary, while the quantitative treatment of intersectionality presents valuable opportunities, it must be undertaken with a commitment to preserving the depth and complexity of the identities and experiences it seeks to represent. Balancing these considerations will be key to advancing intersectionality in a way that remains true to its original purpose while leveraging the benefits of quantitative analysis.

### 2.10. Future research

Future research should focus on advancing the quantitative treatment of intersectionality, moving beyond simply integrating quantitative methods into intersectionality research to developing more refined tools for quantifying the complex interplay of intersecting identities. This involves creating and validating models that capture the interactive and multiplicative effects of multiple social identities—such as race, gender, class, and sexual orientation—on various outcomes.

One promising direction is the development of quantitative scales or indices that represent the degree of marginalization or privilege associated with specific intersections of identities. These could be similar to a summary scale or an interactive and multiplicative model, where the combined impact of identities is greater than the sum of its parts. Such scales could offer a more nuanced understanding of how intersecting identities contribute to disparities in health, socioeconomic status, and other key areas.

Another avenue for future research is the exploration of dynamic models that account for the changing nature of intersectional identities over time and across different contexts. These models would allow for the continuous updating of an individual’s intersectionality score, reflecting shifts in their social, economic, and environmental conditions. This approach would enable researchers and policymakers to better predict and address the evolving needs of individuals as they move through different life stages and contexts.

Finally, future research should aim to create visual and computational tools that can make the quantitative treatment of intersectionality more accessible and actionable for a wider range of stakeholders. For instance, interactive dashboards or visual representations could help policymakers and practitioners better understand the impact of intersecting identities on various outcomes, leading to more informed and equitable decision-making.

By advancing the quantitative treatment of intersectionality, future research can contribute to a deeper, more precise understanding of how intersecting identities shape life experiences, ultimately leading to more effective interventions and policies that address the needs of marginalized groups.

### 2.11. A Note on Quantitative Methods in Intersectionality Research

It is important to clarify that quantitative methods are already integrated into intersectionality research. We are not suggesting that all intersectionality research is qualitative; rather, our focus is on the definition, conceptualization, and treatment of intersectionality itself. The definition of intersectionality is inherently qualitative, emphasizing the complex, overlapping nature of social identities and the ways they interact to shape individual experiences.

By this, we mean that intersectionality itself can be represented quantitatively—a quantitative representation of marginalization due to the intersections of identities. This approach involves creating models that capture the interactive and multiplicative effects of intersecting identities, rather than merely adding up their separate impacts (which would be cumulative and additive). Examples of such quantitative representations could include a horoscope-like diagram or a summary scale that encapsulates the unique and combined effects of multiple identities. These tools could provide a more precise and dynamic understanding of how different aspects of identity interact to produce marginalization, allowing for more accurate predictive models and better-targeted interventions.

In this context, intersectionality becomes not just a qualitative framework but a measurable, quantifiable concept that can be directly applied in research and policy. This shift enables a more detailed analysis of how intersecting identities compound marginalization, thus providing a stronger foundation for addressing the specific needs of individuals within marginalized groups.

## 3. Quantitative Intersectionality Scoring System (QISS)

### 3.1. Introduction to QISS:

The Quantitative Intersectionality Scoring System (QISS) framework is a novel approach designed to quantify intersectional identities and their compounded effects on various outcomes such as health, economic stability, and social mobility. Unlike traditional qualitative approaches to intersectionality, which focus on descriptive analyses of overlapping social identities (e.g., race, gender, class, sexual orientation), QISS provides a systematic, numeric scoring method that can be integrated into predictive models, enhancing their sensitivity and specificity. By converting intersectional identities into quantifiable metrics, QISS allows for a more robust understanding of how these identities interact dynamically within different social and economic contexts. The introduction of QISS enables researchers, policymakers, and service providers to incorporate the complexity of intersectionality into their analyses, allowing for more targeted and equitable decision-making.

### 3.2. Development of QISS:

QISS is developed through a comprehensive framework that assigns numerical values or scores to different social identities and their intersections. This scoring system is based on empirical evidence and statistical modeling, incorporating factors such as race, ethnicity, gender, socioeconomic status, sexual orientation, disability, and other relevant social determinants. Each identity component is weighted according to its documented impact on specific outcomes, such as health disparities, educational attainment, or economic opportunities. For instance, a QISS could assign a higher score to a Black woman from a low-income background with a disability, reflecting the compounded social disadvantages that may affect her health outcomes compared to a White man from a high-income background without a disability. The scores can be adjusted based on geographic, temporal, and contextual factors, allowing for dynamic and context-specific applications.

### 3.3. Methodological Approaches for Calculating QISS:

The calculation of QISS involves several methodological steps. First, relevant data is collected to capture the dimensions of intersectionality for a given population, including demographic information, socioeconomic variables, health indicators, and more. Second, statistical techniques such as regression analysis, machine learning, or multi-level modeling are employed to estimate the relative weights of each identity dimension. Third, interaction terms are used to quantify the combined effects of these identities on the outcome of interest. Finally, the scores are standardized and validated using a cross-validation approach to ensure they accurately represent the intersectional effects. Advanced techniques like latent class analysis or structural equation modeling can also be utilized to refine QISS, allowing for even more precise estimates of intersectionality’s impact.

### 3.4. Applications of QISS in Predictive Modeling and Policy Development:

The QISS framework offers significant opportunities for enhancing the predictive utility of models in various domains, including health, education, and social policy. By incorporating QISS into predictive models, researchers can better account for the non-linear and interactive effects of multiple social identities on outcomes. For example, in health research, QISS can help identify which intersectional groups are most at risk for certain diseases, such as cardiovascular disease or mental health disorders, based on their compounded social disadvantages. In policy development, QISS can be used to evaluate the potential impacts of policy interventions more accurately by considering how different intersectional groups might respond. For instance, a policy aimed at improving access to healthcare services might have varying effectiveness among different groups based on their QISS, thereby helping to optimize resource allocation and program design.

### 3.5. Potential Challenges and Future Directions for QISS Implementation:

While QISS represents a promising advance in the quantitative analysis of intersectionality, several challenges must be addressed for its effective implementation. One challenge is the availability and quality of data, as comprehensive and representative datasets are essential for accurate QISS calculation. Another challenge is the potential for misinterpretation or misuse of QISS, particularly if the scores are not properly contextualized or if they reinforce stereotypes rather than promote equity. To mitigate these risks, ongoing research is needed to refine the QISS methodology, including developing guidelines for its ethical application. Additionally, interdisciplinary collaboration will be crucial in advancing the QISS framework, ensuring it is grounded in both robust quantitative methods and a deep understanding of intersectional theory.

QISS offers a transformative approach to intersectionality by providing a quantitative, data-driven framework for analyzing how multiple social identities intersect to influence outcomes. By enhancing the precision and utility of predictive models and allowing for more nuanced policy evaluations, QISS has the potential to significantly improve efforts aimed at promoting social justice, health equity, and economic opportunity. As the field continues to evolve, further research and methodological innovation will be essential in realizing the full potential of QISS in both academic and practical applications.

### 3.6. Array Analytical Approach:

When considering the complexities of social identities, an array analytical approach allows us to systematically analyze the interactions between various intersectional identities by representing them in a structured, multi-dimensional format [33]. This approach conceptualizes each identity dimension (e.g., race, gender, class, sexual orientation) as an axis within a multi-dimensional array, where each individual can be located at the intersection of these axes. For example, a two-dimensional array might represent gender on one axis (male, female, non-binary) and race on another (White, Black, Asian, Hispanic, etc.), creating a grid where each cell corresponds to an intersectional identity (e.g., Black non-binary, Asian male). By expanding this model to include additional dimensions—such as socioeconomic status, disability, or sexual orientation—researchers can more comprehensively capture the complexity of lived experiences across diverse populations. This structured approach enables the identification of unique patterns and disparities, such as understanding how Black women from low-income backgrounds might experience mental health outcomes differently from White women in similar economic conditions or Black men with higher income levels. Moreover, an array analytical approach facilitates the inclusion of continuous variables (e.g., income level, age) alongside categorical ones, allowing for more nuanced analyses that consider both gradations within identities and their intersections. By quantifying these intersections, the array analytical approach enhances the ability of researchers to model the specific ways in which social determinants of health or other factors affect different groups, thereby improving the precision of interventions aimed at reducing inequalities.

### 3.7. Factorial Designs:

Factorial designs offer another systematic approach to understanding the interactions between multiple intersectional identities by testing the combined effects of several factors simultaneously [34]. Unlike traditional experimental designs that may focus on a single variable, factorial designs allow for the examination of multiple variables and their interactions, providing a more nuanced understanding of how intersectional identities intersect to influence outcomes. For example, a factorial design could be used to study the combined effects of race (e.g., Black, White), gender (e.g., male, female), and class (e.g., high, low) on health outcomes such as hypertension prevalence or mental health. By assigning participants to various combinations of these factors (e.g., Black male from a low-income background, White female from a high-income background), researchers can evaluate both the main effects of each factor and the interaction effects between them. This approach can reveal, for instance, whether the health disparities faced by low-income Black men are more pronounced than those faced by low-income White men, or whether the intersection of race and class creates unique vulnerabilities for specific groups. Factorial designs also allow for the inclusion of moderating variables, such as age or geographical location, to see how these factors further influence the interactions between primary identities. For policymakers and practitioners, the insights gained from factorial designs can inform more targeted and equitable policy interventions, as they provide empirical evidence of how different social identities intersect and shape lived experiences. This, in turn, enables a more tailored approach to addressing health disparities, educational inequities, or economic disadvantages across different intersectional groups.

Using an array analytical approach to understand the interactions between multiple social identities would indeed require an n×mn \times mn×m array rather than the traditional, overly simplistic 2×22 \times 22×2 array. Traditional 2×22 \times 22×2 designs are limited in their ability to capture the complexity and variability inherent in real-world data, particularly when dealing with intersectionality. For instance, a 2×22 \times 22×2 array might consider only two variables—such as gender (male, female) and race (Black, White)—and the interaction between them. However, this approach falls short in representing the vast diversity of social identities and their intersections. In contrast, an n×mn \times mn×m array allows for the inclusion of numerous categories and subcategories across different dimensions, such as race (e.g., White, Black, Asian, Hispanic, Native American), gender (e.g., male, female, non-binary, genderqueer), sexual orientation (e.g., heterosexual, gay, bisexual, asexual), socioeconomic status (e.g., low, middle, high), disability status, and more. Each of these dimensions would be represented as an axis within the array, creating a grid that captures every possible intersection. (Box 1)

For example, consider an n×mn \times mn×m array where one axis represents various racial and ethnic identities (White, Black, Asian, Hispanic, etc.), and another represents gender identities (male, female, non-binary, etc.), while additional axes capture other social determinants like socioeconomic status (low, middle, high), sexual orientation, and disability status. This multi-dimensional array would create a matrix with multiple layers where each cell corresponds to a unique intersection of these identities, such as a Black, non-binary individual from a low socioeconomic background or an Asian, cisgender male with a high socioeconomic status and a disability. By expanding beyond the 2×22 \times 22×2 array, this approach can account for more complex and realistic scenarios, enabling researchers to analyze how various combinations of identities impact outcomes like health, education, or economic stability. It also allows for more robust modeling of interactions, such as how the intersection of race, gender, and socioeconomic status influences mental health disparities. This higher-dimensional array enables a more comprehensive understanding of how multiple forms of oppression or privilege intersect, providing more accurate insights for developing targeted, equitable policies and interventions. Thus, moving towards an n×mn \times mn×m array structure enhances the granularity and specificity of analyses, making them more relevant for addressing the complexities of intersectional identities in research and policy.

## 4. Conclusion

The concept of quantitative methods into intersectionality research is not just a theoretical advancement—it is a practical necessity for improving the predictive accuracy of our models and the effectiveness of our policies. By assigning scores to intersecting identities, we can better understand and address the complex realities faced by individuals at the intersection of multiple forms of marginalization. This approach will enable us to make more informed decisions, create more equitable policies, and provide targeted support that evolves with individuals’ changing circumstances. Ultimately, quantitative intersectionality offers a pathway to more responsive and effective interventions, ensuring that everyone’s life chances are accurately understood and fairly addressed.

## Box 1.

An example of 3×3 and 3 ×2 matrices (arrays) multiplicate and generate a new 3× 2 array.
